# Case report and surgical video presentation: Combined laparoscopic and cystoscopic partial bladder cystectomy for excision of deeply infiltrating endometriosis

**DOI:** 10.1016/j.amsu.2018.09.038

**Published:** 2018-09-28

**Authors:** Jennifer C. Pontré, Jessica M.A. Yin, Bernadette Brown, Krishnan Karthigasu, Bernadette McElhinney

**Affiliations:** a Endoscopy Unit, King Edward Memorial Hospital, Subiaco, Western Australia, Australia; b Urology and Urogynaecology Unit, King Edward Memorial Hospital, Subiaco, Western Australia, Australia

**Keywords:** Deeply infiltrating endometriosis, Laparoscopy, Cystoscopy, Cystectomy, Case report

## Abstract

**Introduction:**

Whilst endometriosis is a relatively common condition, deeply infiltrating endometriosis (DIE) of the bladder is less so, and when medical treatment fails, surgical management is an effective option. We present a case report and surgical video of a patient undergoing combined laparoscopic and cystoscopic excision of deeply infiltrating endometriosis of the bladder.

**Design:**

Case report (Canadian Task Force Classification III) and step-by-step explanation of the surgery using video. Exemption was granted from the local institutional review board.

**Presentation of case:**

We present a case report and surgical video of a 36-year-old nulliparous patient presenting with a 12-month history of sudden onset cyclical dysuria and haematuria. Imaging demonstrated a deeply infiltrating endometriotic nodule involving the bladder. The patient underwent a combined laparoscopic and cystoscopic excision of deeply infiltrating endometriosis of the bladder. The procedure was uneventful and the patient progressed to a full recovery.

**Discussion:**

DIE is a highly invasive form of endometriosis which is defined arbitrarily as endometriosis infiltrating beneath the peritoneum by 5mm or greater. When medical therapy is declined or fails, surgical excision by partial cystectomy would appear to be the most effective management option. A combination of cystoscopy and laparoscopy has been shown to be a safe and feasible procedure, with a low rate of complications. It represents the ideal way by which to identify the resection limits for complete excision of the lesion, and allows for optimal repair of the bladder defect.

**Conclusion:**

Combined laparoscopic and cystoscopic partial cystectomy for excision of deeply infiltrating bladder endometriosis is a safe and feasible procedure in our institution.

## Highlights

• A case of surgical management of deeply infiltrating endometriosis of the bladder is reported.
• Combined cystoscopic and laparoscopic excision can be performed for the treatment of this form of endometriosis.
• This approach is safe, feasible and has a low rate of complications in the current literature.

## 1 Introduction

Whilst endometriosis is a relatively common condition, deeply infiltrating endometriosis (DIE) of the bladder is unusual. Many dedicated Endometriosis Centres worldwide have limited surgical experience in the treatment of this type of endometriosis. The ideal approach involves a combination of cystoscopic assessment of the bladder, and laparoscopy. This combined approach allows the lesion to be adequately delineated and fully excised. We present a case report and surgical video of a patient undergoing combined laparoscopic and cystoscopic excision of deeply infiltrating endometriosis of the bladder.

## 2 Design

Case report (Canadian Task Force Classification III) and step-by-step explanation of the surgery using video. Exemption was granted from the local institutional review board. This work has been reported in line with the SCARE criteria [1].

## 3 Presentation of Case

A 36-year-old, para 0 presented with a 12-month history of sudden onset cyclical dysuria and haematuria, on a background of longstanding dysmenorrhoea and dyschezia. A tertiary level transvaginal ultrasound scan demonstrated adenomyosis, tethered ovaries and a 25mm endometriotic bladder nodule invading through the bladder wall. Concurrent MRI confirmed a 25mm heterogeneous mass postero-superiorly within the bladder. The upper renal tracts were normal.

The patient had no other medical or surgical history, was not on regular medications, and had no relevant genetic, family or psychosocial history. All medical treatments for endometriosis are contraceptive, and as she was hoping to conceive in the short term, the patient declined medical management and elected to undergo surgical management as first line therapy. Following detailed multi-disciplinary discussion and informed consent, she underwent combined cystoscopic and laparoscopic excision of the bladder nodule. The procedure was performed by a consultant gynaecologist, gynaecology fellow and consultant urologist. At the commencement of the procedure, the urologist marked the extent of the lesion with diathermy via the cystoscope. The gynaecologists reflected the bladder from the lower uterine segment and excised the nodule laparoscopically using the internal markings as a guide to the limit of the lesion. The defect was sutured laparoscopically in one layer using multi-filament, absorbable suture.

The procedure and post-operative course were uneventful and discharge occurred on day 4. An indwelling catheter was left in situ for 4 weeks. A cystogram was performed to exclude a defect in the repair prior to removal. At post-operative review at 8 weeks, the patient described complete resolution of urinary symptoms, and was highly satisfied with the surgery and outcome. Histopathological analysis of the excised bladder nodule confirmed endometriosis.

## 4 Description of surgical steps demonstrated in surgical video

Prior to laparoscopy, ureteric JJ stents were placed. At laparoscopy, on initial inspection of the pelvis, tethering of the left ovary and fallopian tube and a 2 cm endometrioma attached to the retrocervix were noted. The left ovary and fallopian tube were mobilised and the endometrioma excised. Deeply infiltrating endometriosis tethering the rectosigmoid to the posterior right vault was noted, however not surgically excised as the patient had expressed a desire to avoid bowel surgery. The right ovary and tube were noted to be normal and mobile. Both ureters were identified. Anteriorly the bladder nodule was visualised and palpated. Cystoscopy was simultaneously performed. The nodule was noted to be 3cm in size and central in the bladder base, but closer to the left ureteric orifice. The bladder was reflected from the uterine isthmus; a McCartney tube in situ helped delineate the anatomical planes. Cystoscopically, a ‘Colling's’ knife was used to outline the nodule by scoring into the bladder mucosa and incising into, but not completely through, the detrusor muscle. The nodule was completely excised laparoscopically. The left and right angles of the cystotomy were secured and the defect was closed with a single layer of 2.0 vicryl. The integrity of the repair was confirmed by repeat cystoscopy.

## 5 Discussion

DIE is a highly invasive form of endometriosis which is defined arbitrarily as endometriosis infiltrating beneath the peritoneum by more than 5mm. When the disease involves the bladder, the endometriotic lesion can infiltrate the detrusor muscle to partial or full thickness. When medical therapy is declined or fails, surgical excision by partial cystectomy would appear to be the most effective management option [2,3]. A combination of cystoscopy and laparoscopy is a safe and feasible procedure, with a low rate of complications reported in the current literature [[4], [5], [6], [7]]. It represents the ideal way in which to identify the resection limits for complete excision of the lesion and allows for optimal repair of the bladder defect [8,9], and is superior to the alternative option of excision via laparoscopy alone as it allows better delineation of the lesion. It is of course important to counsel the patient preoperatively and engage appropriately trained personnel. As this particular form of combined surgery is not performed in a majority of centres around Australia, this case report adds to the current literature on the subject. The presentation of the technique via video is invaluable in the teaching and demonstration of the method.

## 6 Conclusion

Laparoscopic and cystoscopic partial cystectomy for excision of deeply infiltrating bladder endometriosis is employed increasingly for the treatment of this form of the disease. We conclude that it is a safe and feasible procedure within our institution.

## Ethical approval

Exemption was granted from the local institutional review board.

## Sources of funding

No funding was sought for this research.

## Author contribution

JP performed the operation, collected data from medical records, wrote the manuscript and edited the video. JY, BB, KK and BM assisted at the operation and assisted in the writing and editing of the manuscript and video. All authors contributed in the critical revision of the manuscript. All authors have read and approved the final manuscript.

## Conflicts of interest

All authors have no conflicts of interestSources of funding.

## Registration registeration number

N/A.

## Guarantor

Jennifer Pontre.

### Consent

Informed consent was obtained from the patient for this publication.

## Appendix A Supplementary data

CARE Guideline Checklist CARE Guideline Checklist

AMS Cystectomy.mp4 AMS Cystectomy.mp4

IJSCR Cystectomy.mp4 IJSCR Cystectomy.mp4
